# Secular Trends in Pubertal Growth Acceleration in Swedish Boys Born From 1947 to 1996

**DOI:** 10.1001/jamapediatrics.2019.2315

**Published:** 2019-07-22

**Authors:** Claes Ohlsson, Maria Bygdell, Jimmy Celind, Arvid Sondén, Anders Tidblad, Lars Sävendahl, Jenny M. Kindblom

**Affiliations:** 1 Centre for Bone and Arthritis Research, Department of Internal Medicine and Clinical Nutrition, Institute of Medicine, the Sahlgrenska Academy at University of Gothenburg, Gothenburg, Sweden; 2 Bioinformatics Core Facility, Sahlgrenska Academy at University of Gothenburg, Gothenburg, Sweden; 3 Department of Women’s and Children’s Health, Karolinska Institutet, and Pediatric Endocrinology Unit, Karolinska University Hospital, Stockholm, Sweden

## Abstract

**Importance:**

A secular trend for earlier menarcheal age has been established in girls but there are few studies of pubertal timing for boys.

**Objective:**

To determine if there is a secular trend for earlier pubertal timing among boys.

**Design, Setting, and Participants:**

For this population-based retrospective cohort study conducted in Gothenburg, Sweden, we collected heights and weights from school health records for boys born consecutively from January 1 and onwards in 1947 and every 5 years from 1951 to 1996 (n = 375 for each birth cohort from 1947-1991, n = 340 for the birth cohort in 1996, and n = 4090 for the total cohort). We estimated age at the peak height velocity (PHV), the maximum growth velocity during puberty, and childhood body mass index (BMI) at age 8 years for all study participants. The data were analyzed during 2018 and 2019. Boys were eligible if they had a complete personal identity number and data to calculate their age at PHV and childhood BMI. Approximately 2.4% of the original study population was excluded because they lacked a personal identity number, and in the remaining study population, 4090 (69%) had sufficient data to calculate childhood BMI and age at PHV.

**Exposures:**

The exposure was birth year and a potential confounding factor was childhood BMI.

**Main Outcomes and Measures:**

The outcome was age at PHV.

**Results:**

Of the 4090 participants, most were white and the mean (SD) age at PHV was 13.9 (1.1) years. A linear regression model revealed a significant association between year of birth and age at PHV. Age at PHV was 1.5 months earlier for every decade increase in birth year (95% CI, −1.72 to −1.19; *P* < .001). After adjusting for childhood BMI, age at PHV was 1.2 months earlier per decade increase in birth year (95% CI, −1.41 to −0.89). All analyses were repeated in the subgroup of boys born in Sweden and with parents born in Sweden with similar results, indicating that the secular trend was not explained by demographic changes in the population between 1947 and 1996.

**Conclusions and Relevance:**

We provide evidence of a secular trend for earlier pubertal timing in boys that is partially explained by an increased childhood BMI, but other factors that are unknown contribute.

## Key Points

**Question:**

Is there a similar secular trend of earlier pubertal timing among boys as with girls?

**Findings:**

In this cohort study that included 4090 boys and spanned 50 years, there is a secular trend for earlier pubertal timing among boys in Sweden that is independent of increased childhood body mass index during the same period.

**Meaning:**

The secular trend of earlier pubertal timing in boys is partially explained by increasing childhood body mass index, but other factors that are still unknown also contribute.

## Teaser

This cohort study examines secular trends for earlier pubertal timing in Swedish boys born from 1947 to 1996 using age at peak height velocity as objective assessment of pubertal timing.

## Introduction

Puberty, the transition from childhood to adulthood that ends with attaining reproductive capability, is a milestone in human development. The pubertal process consists of a series of events that can be used to determine the timing of puberty. A physical examination of adolescent patients, and estimation of either breast development or testicular volume according to Tanner stages, is often used in routine pediatric care.^1^ Self-reported pubertal demarcations can be used to estimate pubertal timing retrospectively. Age at menarche in girls has substantial accuracy, whereas for boys there is no reliable corresponding pubertal event.^2^ Studies on male pubertal timing are therefore scarce. If several measurements of height during pubertal growth are available, the timing of the pubertal growth spurt (age at the peak height velocity [PHV]) can be used as an objective assessment of pubertal timing. Age at PHV is the age at the maximum growth velocity during puberty and occurs approximately 2 years and 1.5 years after pubertal onset in boys and girls, respectively.^3^

A secular trend (ie, not a cyclical or seasonal trend, but a trend over a long period) of earlier menarcheal age has been observed in Europe and the United States since the mid-19th century,^4^ partly due to increased childhood body mass index (BMI; calculated as weight in kilograms divided by height in meters squared) during the last 60 years.^5^ Early menarche has been associated with an increased risk of diseases, such as type 2 diabetes and breast cancer.^6,7^ These findings indicate that changes in menarcheal age might have implications for health and disease later in life. To our knowledge, for boys, no large population-based study has been performed that covers a long time span with an objective assessment of pubertal timing that captures the entire pubertal period and with adjustment for childhood BMI.

Whether there is a secular trend for earlier pubertal timing in boys is not established and thus represents a knowledge gap regarding male puberty.^8^ We have previously developed a method for objectively assessing pubertal timing based on the height growth curve.^9^ Using this method, we recently demonstrated that childhood BMI is inversely associated with age at PHV in boys with normal weight but not in boys with overweight in a large population-based cohort of 31 971 boys.^10^ Moreover, we have reported a pronounced increase in mean childhood BMI among 8-year-old boys from the 1940s until now.^11^ Here, we address the earlier mentioned knowledge gap regarding male pubertal timing and hypothesize that there is a secular trend for earlier pubertal timing in boys that can be partially explained by childhood BMI. This study’s aim was to investigate the association between birth year and changes in male pubertal timing independent of changes in childhood BMI. To this end, we used the population-based BMI Epidemiology Study (BEST) cohort covering 50 years of growth data in men born from 1947 to 1996 with age at PHV available.

## Participants and Methods

### Data Collection and Study Population

The population-based BEST cohort was initiated with the overall aim to determine the role of childhood obesity and pubertal timing for various diseases in adult life.^12,13,14^ The BEST cohort included individuals who completed school in Gothenburg municipality, Sweden, and afterwards had their school health record stored in the Archives of City of Gothenburg and Region Västra Götaland. The school health records include data on height and weight as measured by specially trained school nurses. For height measurements, a wall-mounted stadiometer was used and the pupils were weighed wearing lightweight clothing. These health examinations were performed according to a prespecified program throughout childhood and until the children finished secondary school and include all children in Sweden (98.5% for school health care from calendar year 1952).^15^ The data from regular health visits at school health care were noted in school health care records and have been retrieved from these records for use in research.^15^ In addition to these childhood data, height and weight have also been retrieved from the conscription register. At military conscription, which was mandatory in Sweden from 1901 to 2010,^16^ all recruits were examined and had their height measured by specially trained staff using a wall-mounted stadiometer. To determine age at PHV for individuals who lacked final height measurements from school health records or the conscription register, we retrieved heights from the passport register, which includes self-reported heights for all individuals holding a passport in Sweden (eMethods in the Supplement). The ethics committee at the University of Gothenburg approved the study and waived the requirement for written informed consent.

### Linkage With Registers From Statistics Sweden

Using the individuals’ personal identity numbers (PINs), the BEST cohort was linked with the Longitudinal Integration Database for Health Insurance and Labor Market Studies at Statistics Sweden. The country of birth for each individual and his parents was also retrieved.

### Curve Fitting and Statistical Analyses

To adequately calculate age at PHV in an unbiased manner, height measurements before, during, and after the pubertal period are required. We calculated age at PHV according to a modified infancy-childhood-puberty model^17^ as previously described.^9^ For each growth curve with sufficient information, we used the nls.lm function in the R package minpack.lm (R Foundation). The model was fitted by minimizing the sum of squares using a modification of the Levenberg-Marquardt algorithm and it also had tests controlling for convergence. A good model fitting was confirmed through visually inspecting all curves (M.B. and J.M.K.). Age at PHV was defined as the age at the maximum growth velocity during puberty and was estimated by the model. Childhood BMI at age 8 years was calculated for every included individual using all paired height and weight measurements between age 6.5 and 9.5 years. This age interval was selected to represent the childhood period after infancy but before the confounding association of puberty with body composition and BMI in boys. Because BMI is also age dependent within the selected childhood period, age adjustment of BMI within the interval was performed using a linear model with BMI as a dependent variable and age as an independent variable. For every BMI measurement within the interval, this linear assumption was used to estimate a BMI at exactly age 8 years. R statistical software, version 3.4.2 (R Foundation), was used to calculate childhood BMI and age at PHV.^18^

Descriptive statistics for age at PHV and childhood BMI were calculated for each birth cohort. The overall trend was calculated using a linear regression and the comparison between mean age at PHV for the different birth cohorts was tested using 1-way analysis of variance followed by the Tukey post hoc test. The distribution of age at PHV for the different birth cohorts was described using the 5th, 25th, 50th, 75th, and 95th percentiles. The association between childhood BMI and age at PHV has previously been shown to be nonlinear.^10^ The nonlinear association between childhood BMI and age at PHV in this cohort was evaluated using a piecewise linear regression model as previously described.^10^ Furthermore, given the nonlinear association between childhood BMI and age at PHV, childhood BMI and quadratic childhood BMI were included when the linear regression model was adjusted for childhood BMI. In all analyses when suitable, log-transformed and standardized childhood BMI was used. A possible nonlinear association between birth year and age at PHV was tested by including a quadratic or cubic term for the birth year. Moreover, we performed a goodness-of-fit test (*F* test) to evaluate if the overall model fit was improved when including the quadratic or cubic terms. For all statistical analyses, SPSS (version 24; IBM) was used, and statistical significance was set at *P *< .05.

## Results

We included 11 male birth cohorts born in 1947 (reference cohort) and every 5 years from 1951 to 1996 (n = 375 for each birth cohort from 1947-1991, n = 340 for birth cohort in 1996, and a total n = 4090 [69% of eligible individuals]) in the present BEST subcohort (eFigure 1 in the Supplement). The mean (SD) age at PHV was 13.9 (1.1) years for the total cohort (eTable 1 in the Supplement).

**Figure 1.**
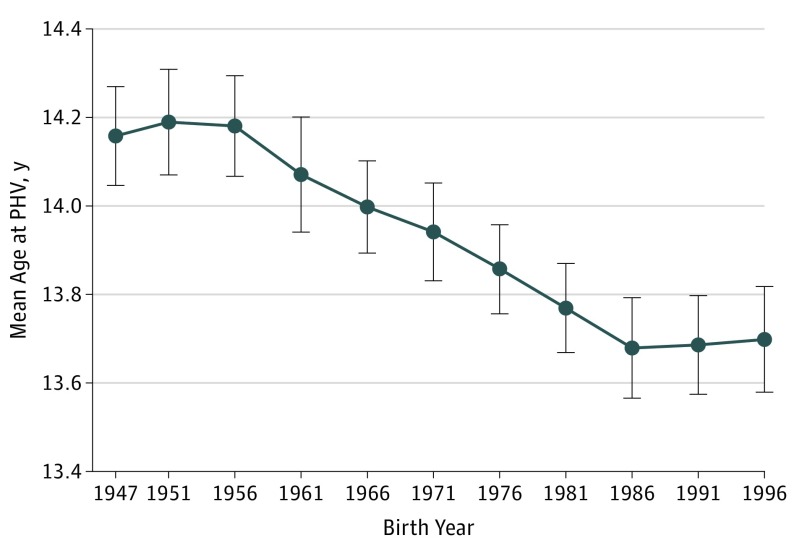
Mean Age at Peak Height Velocity (PHV) for Boys Included in the BMI Epidemiology Study Cohort Born From 1947 to 1996 Values are presented as mean (95% CI), and statistically significant differences vs the birth cohort in 1947 were observed from the birth cohort in 1976 and after (*P *< .01). The *P *for trend is <.001.

**Figure 2.**
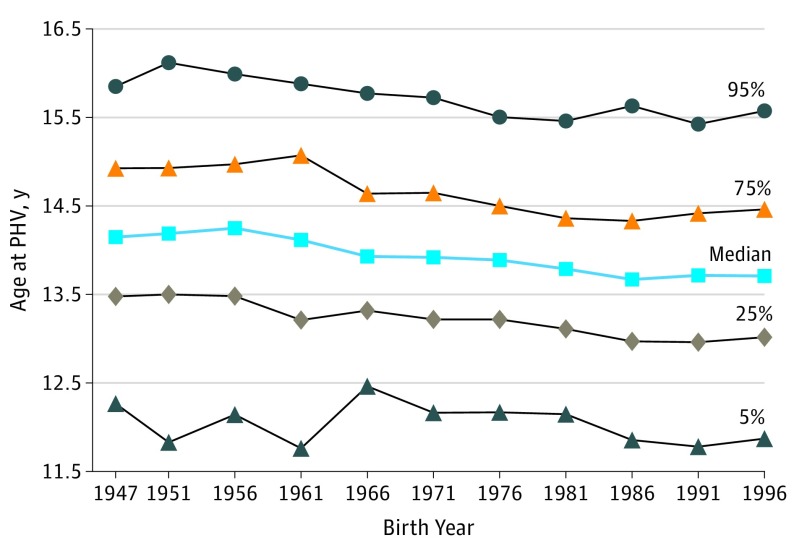
Distribution of Age at Peak Height Velocity (PHV) for Boys Included in the BMI Epidemiology Study Cohort Born From 1947 to 1996

A linear regression analysis between birth year and age at PHV revealed that age at PHV was 1.5 months earlier per decade increase during the study period (−0.12 years per decade increase in birth year; 95% CI, −0.14 to −0.10). The decrease in age at PHV was statistically significant from the birth cohort in 1976 and onwards compared with the birth cohort in 1947 (Figure 1). We did not detect a significant nonlinearity as assessed by including a quadratic or a cubic term for birth year in the regression analysis (*P *values were nonsignificant for the quadratic and cubic terms). Furthermore, goodness-of-fit tests demonstrated that neither adding a quadratic nor a cubic term to the model significantly improved the overall model fit compared with the linear model. The secular trend for age at pubertal timing is shown across the distribution of age at PHV in Figure 2. In subanalyses, linear regression analyses were performed for the highest and lowest percentiles of age at PHV, demonstrating a secular trend for late and early percentiles of age at PHV that was similar for the entire population (eTable 2 in the Supplement). Compared with the entire population, there was a slightly less pronounced secular trend for those with an early age at PHV (eTable 2 in the Supplement). In addition, when age at PHV was displayed according to the participants’ BMI status at age 8 years, the secular trend was observed for participants with normal weight and overweight (eFigure 2 in the Supplement).

**Figure 3.**
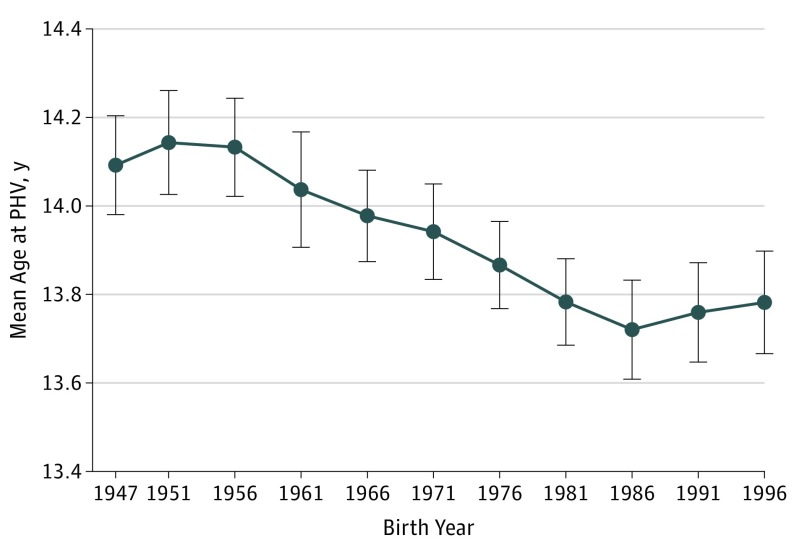
Mean Age at Peak Height Velocity (PHV) Adjusted for Childhood Body Mass Index (BMI) for Boys Included in the BMI Epidemiology Study Cohort Born From 1947 to 1996 Values are presented as mean (95% CI). The *P *for trend is <.001.

There was an inverse association between childhood BMI and age at PHV in the present study population born 1947 to 1996 below, but not above, a threshold at 17.71 (eFigure 3 in the Supplement). Given that childhood BMI increased during the same period,^11^ we next wanted to determine the secular trend of earlier age at PHV independent of childhood BMI*.* When we included age at PHV and childhood BMI (median, 15.8; range 12.3-29.2) in the same linear regression model, we found that the association between birth year and age at PHV was slightly attenuated but remained significant; the age at PHV was 1.2 months earlier per decade increase during the study period (−0.10 years per decade increase in birth year; 95% CI, −0.12 to −0.07; Figure 3).

**Figure 4.**
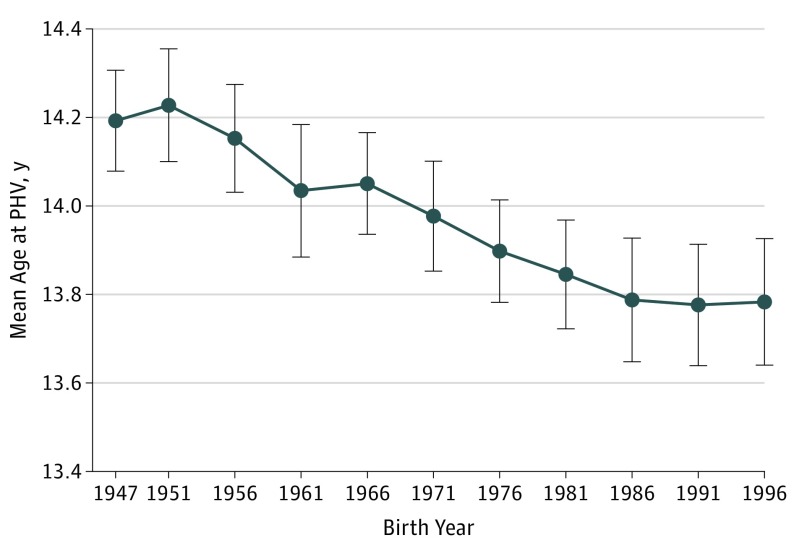
Mean Age at Peak Height Velocity (PHV) Among a Subgroup of Boys Born From 1947 to 1996 in Sweden and With Parents Born in Sweden Values are presented as mean (95% CI). The *P *for trend is <.001.

To investigate a possible confounding effect by demographic changes in the population from 1947 to 1996, we performed a subanalysis including only boys born in Sweden and with parents born in Sweden (3087 [75%] of the study cohort). The subanalysis demonstrated similar results as the main analysis: age at PHV was 1.2 months earlier for every decade increase in birth year (−0.10 years per decade increase in birth year, 95% CI, −0.13 to −0.08; Figure 4). Furthermore, age at PHV was 0.9 months earlier for every decade increase in birth year independent of childhood BMI (−0.07 years per decade increase in birth year; 95% CI, −0.10 to −0.05). Similar to the total cohort, there was no indication of a nonlinear association in the subanalysis that included only boys born in Sweden and with parents born in Sweden.

Thus, there was a clear secular trend for earlier pubertal timing in Swedish boys born from 1947 to 1996 that was independent of prepubertal BMI. Similar results were seen in subanalyses that included only boys born in Sweden and with parents born in Sweden.

## Discussion

In this article, we present evidence of a secular trend for earlier pubertal timing in boys using age at PHV as objective assessment of pubertal timing in 4090 boys in 11 birth cohorts born from 1947 to 1996. Age at PHV was 1.5 months earlier for every decade increase in birth year and 1.2 months earlier per decade after adjusting for childhood BMI at age 8 years.

The concept of a secular trend for earlier pubertal timing and the contribution of increasing childhood BMI to the secular trend in pubertal timing is well established for girls.^8^ However, for boys, studies are scarce and, to our knowledge, it is not established whether age at pubertal timing has changed over time.^8^ Whereas menarcheal age for girls is often easily available, a corresponding self-reported, valid marker for pubertal timing in boys is lacking.

A Danish study used growth data to investigate the change in pubertal timing for boys born from 1935 to 1969. They found that age at PHV was 0.3 years earlier in boys born from 1965 to 1970 compared with boys born from 1935 to 1939.^19,20^ However, the study had several weaknesses. Only 10% of the study population born from 1935 to 1970 was included and for the birth years 1935 to 1939, only 0.2% were included. As the height growth data in that study (from age 7 to 15 years) did not cover the entire pubertal period, boys with late puberty, not completed at age 15 years, were not included. The study did not assess the extent of the secular trend independent of childhood BMI. Moreover, information on country of birth was not available and therefore it is unclear to what degree the secular trend was explained by increasing childhood BMI or confounded by demographic shifts in the population. Another article comparing the average pubertal timing, determined using Tanner staging of secondary sex characteristics, between several different studies that were mostly cross-sectional and collected at different points, indicated that there might be a possible trend for earlier pubertal timing in boys. However, the methods for determining pubertal timing and the composition of the cohorts with regard to recruitment and ethnicity differed substantially.^21^ An independent, well-powered cross-sectional study (n = 826) using secondary sex characteristics failed to detect any evidence of a secular trend.^22^ Thus, a secular trend toward earlier pubertal timing in boys is not as well documented as in girls.

We have previously demonstrated an increase in mean BMI and in the prevalence of overweight among 8-year-old boys born between 1946 and 2006 and an inverse association between BMI at age 8 years and pubertal timing in boys with normal weight but not overweight in 2 cohorts, one of which was born before the obesity epidemic and one that was born late during the obesity epidemic.^10,11^ Here we demonstrate an inverse association between childhood BMI and pubertal timing in the present study population, covering the period from the 1940s to the 1990s. Given our findings in boys regarding a strong inverse association between childhood BMI and pubertal timing and similar previous findings in girls,^23^ it is plausible that the increasing childhood BMI has contributed to the secular trend. It is therefore relevant to determine the secular trend in pubertal timing independent of prepubertal childhood BMI. When we adjusted our analysis on the association between birth year and pubertal timing for childhood BMI, we found that prepubertal childhood BMI contributes to this association but that a substantial part of the association between birth year and age at PHV was independent of childhood BMI. Moreover, a recent study actually demonstrated earlier pubertal timing in boys with overweight than in boys with obesity.^24^ In addition, we were also able to show a significant association between birth year and pubertal timing in a subgroup of men born in Sweden and with parents born in Sweden and thereby confirm that the change in pubertal timing was not explained by demographic changes in the study population between 1947 and 1996. Although the general appearance of the curve indicates that the secular trend mainly happened from birth year 1956 to birth year 1986, we did not detect any statistically significant nonlinearity.

Some studies have suggested an increased use of endocrine disrupting chemicals (EDCs) to account for the secular trend in earlier pubertal timing in girls.^25^ The finding that female mice exposed to the estrogenic acting endocrine disruptor bisphenol A (BPA) prenatally displayed earlier sexual maturation^26^ lends some support to this hypothesis in girls. Boys with moderate exposure to the BPA had earlier pubertal timing compared with boys with the least exposure to BPA,^27^ and urine BPA levels were also inversely associated with pubertal height gain.^28^ Conversely, phthalates have antiandrogenic and obesogenic properties and high urinary phthalates were associated with late pubarche in girls^29^ but were not associated with pubertal onset or levels of serum testosterone in boys.^30^ Thus, EDCs with estrogenic, antiandrogenic, or obesogenic actions may have differing associations with puberty in boys and girls.^27,31,32^ To what extent EDCs contribute to the secular trend in male pubertal timing presented in this study is not clear. Our findings indicate that there is a robust secular trend for earlier pubertal timing in boys that is explained by other unknown factors than the obesity epidemic and demographic changes. Possible other factors of importance might include EDCs, overall better psychosocial environment, and improved nutrition as well as improved health care.

Pubertal timing can be interpreted as a measure of exposure to sex steroids. In girls, early pubertal timing is associated with an increased risk for type 2 diabetes and breast cancer.^6,7^ Whether the secular trend for pubertal timing has implications for future health and disease in men represents a knowledge gap that will be important to address.

### Strengths and Limitations

The strengths of this population-based study include the long study period with data on pubertal timing from 11 birth cohorts during 50 years that allowed us to distinguish between temporary changes and persistent trends. With information on childhood BMI available for the boys in the cohort, we were able to determine that the secular trend was robust and only partially explained by increasing childhood BMI. In addition, we demonstrated that the results are maintained when only participants born in Sweden and with parents born in Sweden were included. Furthermore, we used an objective measurement of pubertal timing in boys. This study’s limitations were that psychosocial and socioeconomic factors were not available. Moreover, we cannot completely exclude the possibility that there is a secular trend in the interval between the onset of puberty and the growth acceleration so that the onset of puberty is unaltered despite earlier growth acceleration. Because of the retrospective design of this study, information on secondary sex characteristics is not available. However, age at PHV, although a surrogate marker for pubertal timing, is derived from direct measurements of height, producing an objective estimate of pubertal timing. Age at PHV shows a strong association with pubertal timing retrieved from detailed longitudinal physical examinations of secondary sex characteristics.^33^ In contrast, self-reported pubertal timing in men displays a modest correlation with secondary sex characteristics as determined by a physician.^34^ In addition, if repeated height measurements are available, age at PHV can, in contrast to detailed longitudinal physical examinations of secondary sex characteristics, be easily estimated for many individuals. Another limitation might be that because our population has a narrow range of prepubertal BMI, we cannot eliminate the possibility that our study underestimated the importance of prepubertal BMI on pubertal timing in a contemporary population of boys exposed to the obesity epidemic.

## Conclusions

We provide evidence of a secular trend for the earlier timing of pubertal PHV, a marker of pubertal timing, in Swedish boys. The secular trend of earlier pubertal timing is partially explained by increasing childhood BMI, but other unknown factors also contribute.

## Notes

Supplement. eMethods. eFigure 1. Flow chart of included participants eFigure 2. Age at PHV according to prepubertal BMI status at 8 years of age eFigure 3. Association between childhood BMI and age at PHV eTable 1. Birth cohort descriptives eTable 2. Associations for early and late age at PHV percentiles
