# Supplementary material for: Secular Trends in Pubertal Growth Acceleration in Swedish Boys Born From 1947 to 1996
Source: JAMA Pediatr. 2019 Jul 22;173(9):860–5. doi: 10.1001/jamapediatrics.2019.2315 (PMC6647355; doi:10.1001/jamapediatrics.2019.2315)
Supplement: Supplement. — eMethods. eFigure 1. Flow chart of included participants eFigure 2. Age at PHV according to prepubertal BMI status at 8 years of age eFigure 3. Association between childhood BMI and age at PHV eTable 1. Birth cohort descriptives eTable 2. Associations for early and late age at PHV percentiles [file jamapediatr-173-860-s001.pdf]

Supplementary Online Content

Ohlsson C, Bygdell M, Celind J, et al. Secular trends in pubertal growth acceleration in Swedish boys born from 1947 to 1996. *JAMA Pediatr*. Published online July 22, 2019. doi:10.1001/jamapediatrics.2019.2315

eMethods.
eFigure 1. Flow chart of included participants
eFigure 2. Age at PHV according to prepubertal BMI status at 8 years of age
eFigure 3. Association between childhood BMI and age at PHV
eTable 1. Birth cohort descriptives
eTable 2. Associations for early and late age at PHV percentiles

This supplementary material has been provided by the authors to give readers additional information about their work.

© 2019 Ohlsson C et al. *JAMA Pediatrics.*

eMethods

Cohort and representativeness

Data collection and validation in the BEST cohort is ongoing. The present BEST sub-cohort included boys born consecutively from the 1st of January and onwards to a total of 375 boys per birth cohort for 1947 and every five years from 1951 to 1991, and to a total of 340 boys for birth cohort 1996 (total n=4090). Birth year 1947 was the first year with a representative number of individuals with information on age at PHV, and is used as the reference year. Since the data collection is not completed, birth year 1996 had a lower number of individuals with height and weight data available than other years. Individuals were eligible for the present study if they had a complete Personal Identity Number (PIN) and data for the calculation of age at PHV and childhood BMI. Individuals were excluded if the PIN was missing in the school health record and not possible to retrieve from population registers (2.4% of the original study population was excluded due to lack of PIN). In the remaining study population, 69% had sufficient data for calculation of childhood BMI and age at PHV (eFigure 1). Of subjects with a childhood BMI at 8 years of age available (n=5272), we compared those included (i.e. had age at PHV available, n=4090) and those not included (i.e. did not have age at PHV available, n=1182) with regard to childhood BMI at 8 years of age. The difference in BMI at 8 years of age for the included (mean 16.1 kg/m^2, [SD 1.7]) and the non-included (16.2 kg/m^2 [1.9]) was 0.6% ($p<0.05$). Thus, the difference between included and non-included subjects with regard to childhood BMI was minor.

© 2019 Ohlsson C et al. *JAMA Pediatrics.*

eFigure 1. Flow chart of included participants

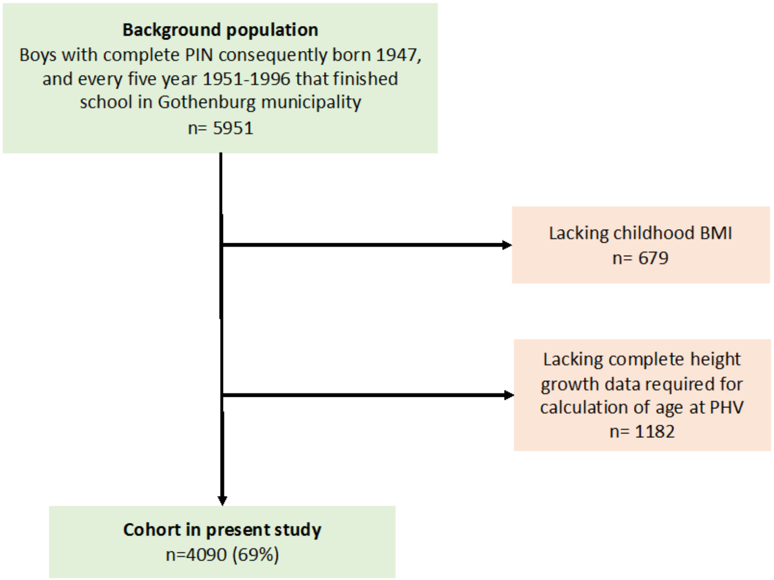

Personal Identity Number (PIN), Body Mass Index (BMI), Peak Height Velocity (PHV).

© 2019 Ohlsson C et al. *JAMA Pediatrics.*

eFigure 2. Age at PHV according to prepubertal BMI status at 8 years of age

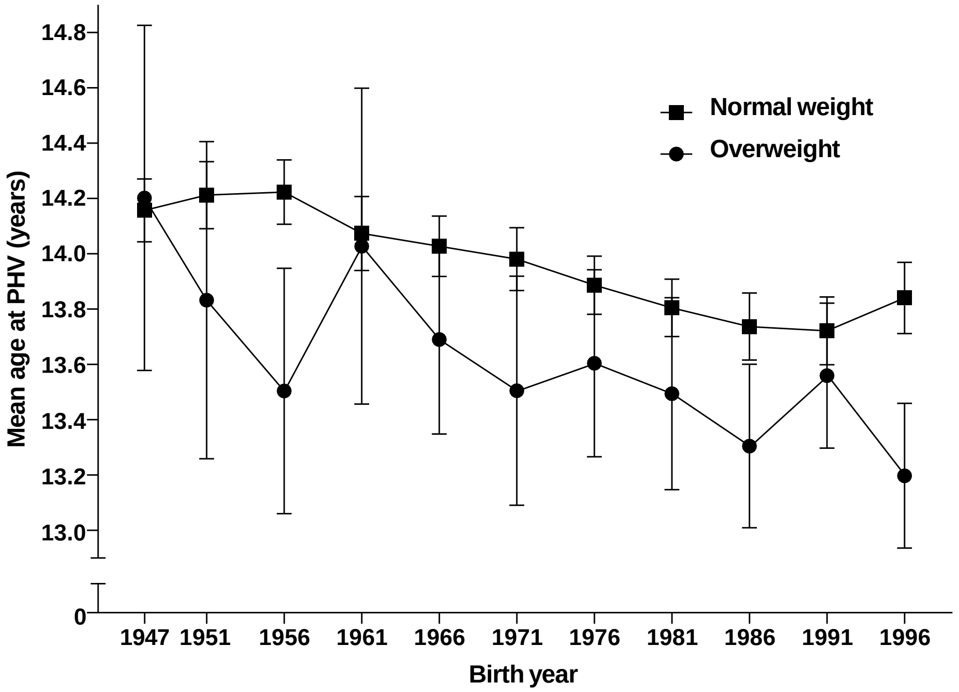

Age at PHV is shown according to normal weight and overweight status at 8 years of age. Overweight includes overweight and obese subjects. Data is shown as mean (95% CI) per year. Linear regressions of birth year versus age at PHV demonstrated a secular trend for normal weight subjects (-0.11 years per decade, 95% CI -0.13;-0.08) and for overweight subjects (-0.13 years per decade, 95% CI -0.20; -0.05).
PHV= Peak Height Velocity, CI = Confidence Interval

© 2019 Ohlsson C et al. *JAMA Pediatrics.*

eFigure 3. Association between childhood BMI and age at PHV

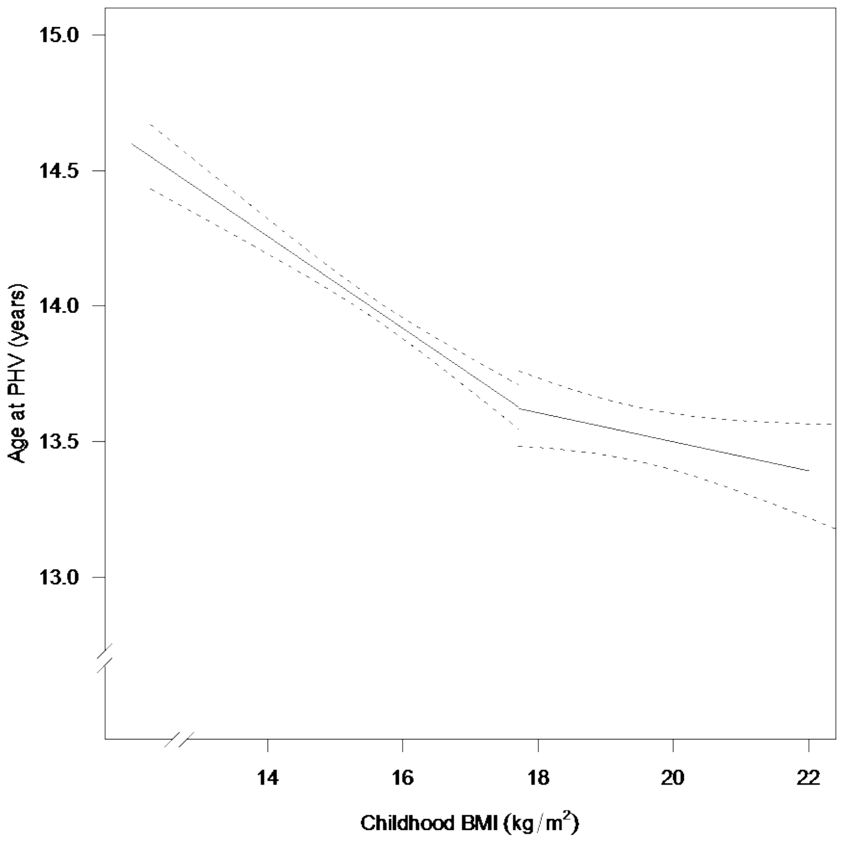

A piecewise linear regression between childhood BMI and age at PHV demonstrated a significant inverse association below a threshold of 17.71 kg/m^2 (-0.17 years per BMI unit, 95% CI -0.20;-0.14), but not above (-0.05 years per BMI unit, 95%CI -0.11;0.002).
BMI= Body Mass Index, PHV= Peak Height Velocity, CI = Confidence Interval

© 2019 Ohlsson C et al. *JAMA Pediatrics.*

eTable 1. Birth cohort descriptives

Birth year	n	Mean (SD) age at PHV (years)
1947	375	14.16 (1.10)
1951	375	14.19 (1.18)
1956	375	14.18 (1.12)
1961	375	14.07 (1.29)
1966	375	14.00 (1.03)
1971	375	13.94 (1.09)
1976	375	13.86 (0.99)
1981	375	13.77 (0.99)
1986	375	13.68 (1.12)
1991	375	13.69 (1.10)
1996	340	13.70 (1.12)

SD=Standard Deviation, PHV=Peak Height Velocity

© 2019 Ohlsson C et al. *JAMA Pediatrics.*

eTable 2. Associations for early and late age at PHV percentiles

Percentiles age at PHV	Months per decade (95% CI)
Entire population	
Percentiles 0-100	-1.46 (-1.19; -1.72)
Early puberty	
Percentiles 0-3	-0.68 (-1.24;-0.12)
Percentiles 0-5	-0.57 (-1.06;-0.08)
Percentiles 0-10	-0.73 (-1.12;-0.34)
Late puberty	
Percentiles 90-100	-1.49 (-1.83;-1.15)
Percentiles 95-100	-1.53 (-1.96;-1.10)
Percentiles 97-100	-1.63 (-2.23;-1.03)

Associations from linear regression analyses for the extremes in age at PHV, given as months/decade with 95% confidence intervals in parentheses. The model has birth year as independent and age at PHV as dependent variable and is unadjusted. PHV= Peak Height Velocity, CI= Confidence Interval.

© 2019 Ohlsson C et al. *JAMA Pediatrics.*
